# New Triterpene Saponins from *Duranta repens* Linn. and Their Cytotoxic Activity 

**DOI:** 10.3390/molecules14051952

**Published:** 2009-05-25

**Authors:** Wafaa S. Ahmed, Mona A. Mohamed, Rabab A. El-Dib, Manal M. Hamed

**Affiliations:** 1 Department of Medicinal Chemistry, Theodor Bilharz Research Institute, P.O.B. 12411, Giza, Egypt; E-mails: eb2003-eg@hotmail.com (W-S.A), manalayman@hotmail.com (M-M.H.); 2 Department of Pharmacognosy, Faculty of Pharmacy, Helwan University, P.O.B. 11795, Ain-Helwan, Cairo, Egypt; E-mail: reldib@yahoo.com (R-A.E-D.)

**Keywords:** *Duranta repens*, triterpene saponins, durantanin IV and V, brine shrimp, HepG2

## Abstract

From the leaves of *Duranta repens* (Verbenaceae) two new triterpene saponins, named durantanin IV (**1**) and V (**2**) were isolated. In addition, ten known compounds were isolated, namely a bidesmosidic saponin, oleanolic acid, three phenylethanoids and five flavonoids. All metabolites were isolated for the first time from this genus except for **3** (oleanolic acid) and **7** (*E/Z* acteoside). The structures were determined mainly by spectroscopic methods (UV, IR, HRESI-MS, ^1^H-, ^13^C-NMR, ^1^H-^1^H COSY, HSQC and HMBC). Cytotoxic screening of the chloroform, ethyl acetate and methanol extracts was carried out on brine shrimps. In addition, the investigated methanol extract and compounds **1**, **2** and **7** showed significant cytotoxic activity against a HepG2 cell line.

## 1. Introduction

The genus *Duranta* comprises about 35 species of evergreen shrubs distributed in tropical and sub-tropical regions. *Duranta repens* Linn. var. *variegata* (syn: *Duranta plumieri* Jacq.) (Verbenaceae) is native to scrub and open woodlands in the West Indies, northern parts of Pakistan and central and South America [1,2,3]. It was introduced to Egypt as an ornamental plant in the 1920s [4]. From the genus *Duranta* several iridoid glycosides such as the durantosides I, II, III, IV and lamiide were isolated [5,6]. The fruits of *D. repens* showed *in vivo* antimalarial activity against *Plasmodium berghei* [7]. Thrombin inhibitory coumarins were isolated from fruits [8]. The ethyl acetate soluble fraction of *D. repens* methanol extract showed antioxidant and antiviral activities [9,10]. Triterpenes [3], flavonoids, steroids, *C*-alkylated flavonoids [11,8], acetosides [10] and some alkaloids [12] were isolated from *D. repens* (var. *variegata*), the most common variety of *D. repens*. In the course of our studies on bioactive constituents from medicinal plants, our preliminary pharmacological screening of the methanolic extract of *D. repens* leaves showed strong cytotoxicity in both a brine shrimp lethality test and a HepG2 cell line. This evidence encouraged the authors to carry out phytochemical studies on this extract. We report herein the isolation and structure elucidation of two new triterpenoidal saponins on the basis of spectroscopic analysis including various two-dimensional (2D) NMR spectroscopic data. Also, cytotoxicity towards brine shrimps was determined for the chloroform, ethyl acetate and methanol extracts of the leaves. The methanol extract and major isolates (**1**, **2** and **7**) were also tested against the HepG2 tumor cell line.

## 2. Results and Discussion

**Figure 1:**
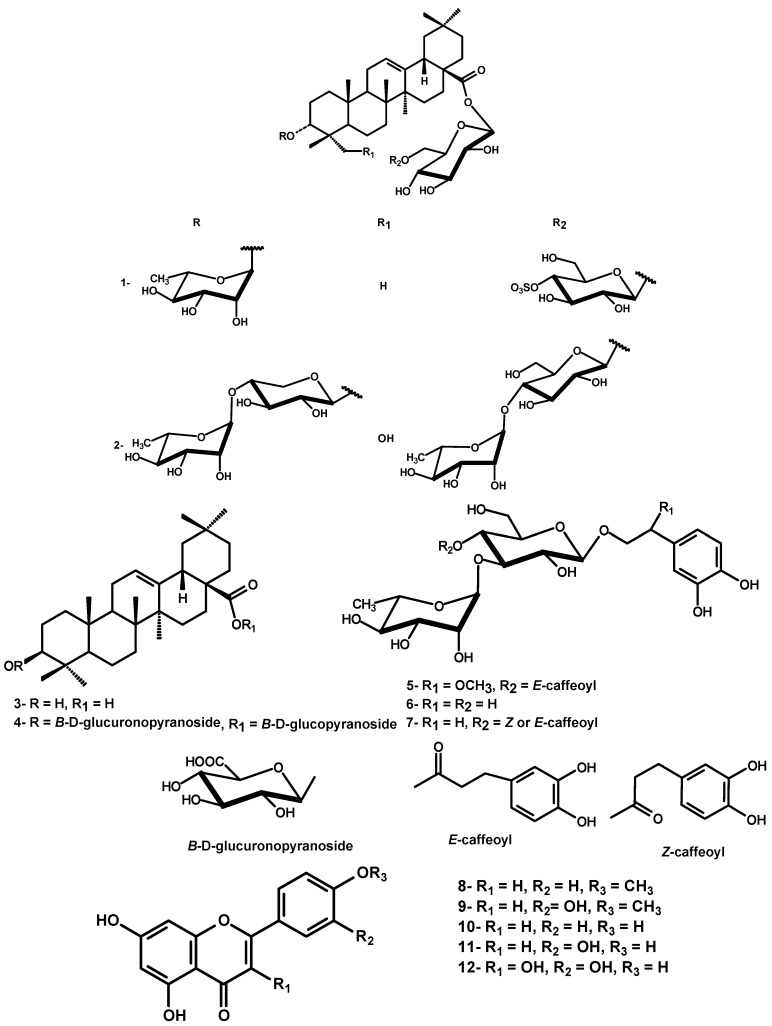
Structure of isolated compounds from the leaves of *Duranta repens.*

The dried leaves of *D. repens* were exhaustively extracted with 85% methanol. Chromatographic separation of the defatted methanol extract over polyamide followed by repeated silica gel and Sephadex LH-20 column chromatography resulted in the isolation of two new triterpenoidal saponins durantanin IV (**1**) and V (**2**), along with oleanolic acid (**3**), the triterpene saponin 3-[(*O*-*β*-^4^C_1_-glucuronopyranosyl)-oxy]olean-12-en-28-oic acid *O*-*β*-D-^4^C_1_-glucopyranosyl ester (**4**) [13], three phenylethanoids, namely campenoside I (**5**) [14], cistanoside E (**6**) [15] and *E*/*Z* acteoside (**7**) [10] and five flavonoids **8-12**, identified as acacetin, diosmetin, apigenin, luteolin and quercetin, respectively [16] (Figure 1). The known compounds were identified by comparison of their physical data with those reported in literature, in addition to comp-PC for phenolic compounds and comp-TLC for triterpenes.

Compound **1**, was isolated as an off-white amorphous powder. The IR spectrum of **1** exhibited a characteristic S-O stretching absorption band at 1,225 cm^-1^. Its molecular formula was established as C_48_H_78_O_20_S by means of HRESI-MS pseudomolecular ion peak at *m/z* 1005.4723 [M-H]^-^ (calcd. 1005.4728). In addition, it gave a diagnostic fragment ion peak at *m/z* 601.4103 [M-H-242-162]^-^ (loss of a sulfohexosyl and hexosyl), followed by 455.3524 [aglycone-H]^-^, corresponding to the loss of a deoxyhexosyl from the last fragment. Mineral acid hydrolysis of **1** afforded glucose and rhamnose in the aqueous phase and its treatment with barium chloride gave a white ppt. of BaSO_4_, confirming the existence of a sulfate moiety [17]. In addition, the alkaline hydrolysis of **1** yielded the prosapogenin, which furnished by further acid hydrolysis rhamnose and oleanolic acid (Comp-TLC and PC with authentic samples). These data together with mass fragmentation, which suggested the location of sulfohexosyl and hexosyl at C-28 due to the presence of diagnostic fragment ion peak at *m/z* 601.4103 [M-H-242-162]^-^ were compatible with a structure of olean 28-sulfoglucosyl-glucosyl ester with an *O*-rhamnoside, most probably at C-3.

The ^13^C-NMR spectrum displayed signals due to seven sp3 methyl carbons at 15.9, 17.4, 17.5, 24.0, 25.2, 28.5 and 31.0, an oxygen-bearing methine carbon at δ 85.1, two olefinic carbons at δ 123.3 and 144.4 and an ester carbonyl carbon at δ 176.0. These data coupled with corresponding information from the ^1^H NMR [7 tertiary methyl proton singlets, hydroxymethine proton at δ 3.95 for H-3, a proton attributed to H-18 axial ring E at δ 3.49 (*dd*, *J* = 13.5, 3.0 Hz) and a broad singlet vinyl proton at δ 5.70 of H-12] confirmed the aglycone moiety as 3-hydroxy-olean-12-en skeleton. The *α*-configuration the OH-3 was determined from the splitting pattern of H-3 (*br s*) and the shielded shift of C-3 at 85.1 compared with that of the *β*-aglycone [18]. The resonances of C-3 at δ 85.1, C-28 at δ 176.0 together with ^1^H NMR signal for *β*-glucopyranosyl ester at 6.50 were characteristic of a bidesmosidic aglycone with 3 α-hydroxyl. All assigned ^1^H and ^13^C-resonances of the aglycone were confirmed by HSQC and HMBC correlation spectroscopy (Table 1). Three anomeric proton signals were assigned at 5.09 (*br s*, *α*-rhamnosyl), 6.50 (*d*, *J* = 8.0, *β*-glucosyl ester), 5.25 (*d*, *J* = 7.8, *β*-glucosyl) through their one bond correlation in HSQC with their own anomeric carbon signals at δ 103.0, 95.9 and 105.1, respectively (Table 1). The sugar moieties were deduced to adopt *α*-^1^C_4_ and *β*-^4^C_1_-pyranose stereostructure in case of rhamnosyl and glucosyl moieties, respectively on the basis of *J*-values of the anomeric protons and δ-values of their ^13^C-resonances (Table 1). The deshielded location of one of the two CH_2_OH glucose carbons at 69.5 suggesting the (1→6) interglycosidic link. Additionally, an unambiguous determination of the sequence and linkage sites was obtained from an HMBC experiment showing cross peak correlations between H-1' (5.09) (rhamnosyl) and C-3 (85.1) aglycone to establish a glycoside moiety at C-3 of the aglycone as 3-*O*-*α*-L-^1^C_4_-rhamnopyranosyl. Similarly, correlations between H-1'' (6.50) glucosyl ester and aglycone C-28 (176.0) and between H-1''' (5.25) glucosyl and primary alcoholic C-6'' (69.5) glucosyl ester were detected to establish a diglucosyl ester moiety at C-28 as 28-*O*-sulfo-*β*-D-^4^C_1_-glucopyranosyl-(1'''→6'')-*O-β*-D-^4^C_1_-gluco-pyranosyl. The strong deshielding of one of the glucosidic protons H-4''' in **1** at δ 4.64, was a strong evidence for the sulfonation of its geminal OH. This evidence was further confirmed by the deshielded shift of C-4''' to 79.0 (∆ + ~ 8 ppm) and shielded shift of both C-3''' and C-5''' relative to those of unsubistituted moiety [19]. The assignment of all ^1^H and ^13^C-resonances was proved by the aid of HSQC and HMBC-correlation peaks and comparison with the corresponding published data for structural related compounds [19,20,21]. Therefore, **1** was confirmed as 3-[(*O*-*α*-L-^1^C_4_-rhamnopyranosyl)-oxy]-olean-12-en-28-oic acid *O*-4'''-*O*-sulfo-*β*-D-^4^C_1_-glucopyranosyl-(1''''→6''')-*O-β*-D-^4^C_1_-gluco-pyranosyl ester (Figure 1). 

Compound **2** was isolated as a white powder. The HRESI-MS of **2** exhibited a pseudomolecular ion peak *m*/*z* 1219.6117 [M^_^H]^-^ suggesting the molecular formula C_59_H_96_O_26_. In addition, it gave diagnostic fragment ion peaks at *m/z* 749.4180 [M-H-146-2x162]^-^ (loss of a rhamnosyl and two hexosyl) and 603.4280 (loss of extra 146 of a second rhamnosyl), followed by 471.4425 [aglycone-H]^-^, corresponding to the loss of a pentoside from the last fragment. Mineral acid hydrolysis afforded glucose, rhamnose and xylose in the aqueous phase. In addition, alkaline hydrolysis of **2** yielded the prosapogenin, which furnished by further acid hydrolysis xylose, rhamnose and hedragenin (Comp-TLC and PC with authentic samples). These data together, with mass fragmentation which suggested the location of two hexosyl and one pentosyl at C-28 due to the presence of diagnostic fragment ion peak at *m/z* 749.4180 [M-H-146-2x162]^-^ were compatible with a structure of hedragenin-rhamnoglucosyl-glucosyl ester with an *O*-rhamnoxyloside moiety, most probably at C-3.

**Figure 2:**
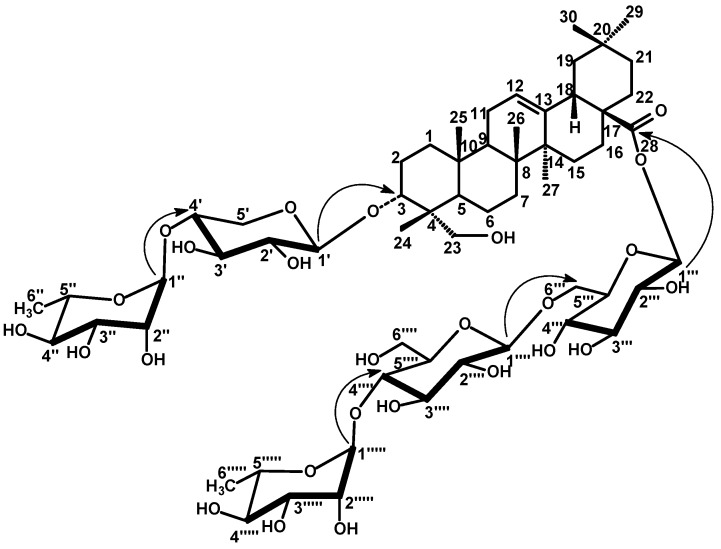
Selected HMBC correlations for compound **2**. Arrows point from H to C.

Compound **2** showed very similar ^1^H- and ^13^C-NMR resonances of the aglycone as those of **1**, except for the shielded location of C-5 at δ 46.9 and C-3 at δ 79.9 to confirm the γ-effect of the carbinol-OH-23 (Table 1). In addition, the Me-23 singlet signal observed in ^1^H-NMR of **1** was replaced by oxymethyl signals at δ 3.75 and 4.11 each (*d*, *J* = 10.5 Hz) in **2**. Similarly, the C-23 methyl signal observed in the ^13^C-NMR spectrum of **1** was replaced by a signal at δ 63.6. The above data indicated that the methyl-23 in **1**, was replaced by a hydroxymethyl group in **2** to confirm the aglycone moiety as 3α,23-dihydroxy-olean-12-en-28-oic acid (hedragenin). The resonances of C-3 at δ 79.9 and C-28 at δ 175.5 together with ^1^H-NMR signal at 6.27 were characteristic of a bidesmosidic hedragenin with 3α-hydroxyl. All assigned ^1^H- and ^13^C-resonances of the aglycone were confirmed by HSQC and HMBC correlation spectroscopy (Table 1). Five anomeric proton signals were assigned at 5.12 (7.5, *β*-xylosyl), 5.86 (*br s*, *α*-rhamnosyl), 6.27 (8.0, *β*-gulcosyl ester), 4.98 (7.8, *β*-glucosyl) and 6.29 (*br s*, *α*-rhamnosyl) confirmed through their direct one bond correlations in HSQC with their own anomeric carbon signals at δ 104.1, 102.5, 95.5, 104.8 and 101.3, respectively, (Table 2). The sugar moieties were deduced to adopt *α*-^1^C_4_- or *β*-^4^C_1_-pyranose stereostructure in case of rhamnosyl and xylosyl or glucosyl moieties, respectively on the basis of *J*-values of the anomeric protons and δ-values of their ^13^C-resonances (Table 2). The interglycosidic and sugar-aglycone linkages were deduced from the long range three bond HMBC correlations. The HMBC (Figure 2) exhibited correlations between H-1' (5.12) xylosyl and C-3 (79.9) aglycone, H-1" (5.86) rhamnosyl and C-4' (78.4) xylosyl to establish a diglycoside moiety at C-3 as 3-*O*-α-L-^1^C_4_-rhamnopyranosyl-(1"→4')-*O*-α-L-^4^C_1_-xylosylpyranoside. Similar, correlations between H-1''' (6.27) glucosyl ester and C-28 (175.5) aglycone, H-1'''' (4.98) glucosyl and C-6''' (69.4) glucosyl ester, H-1''''' (6.29) rhamnosyl and C-4'''' (79.7) of the second glucosyl were detected to establish a triglycosyl ester moiety at C-28 as 28-*O*-α-L-^1^C_4_-rhamno-pyranosyl-(1'''''→4'''')-*O-*β-D-^4^C_1_-glucopyranosyl-(1''''→6''')-*O-*β-D-^4^C_1_-glucopyranosyl. All other ^1^H- and ^13^C-resonances were assigned by the aid of HSQC and HMBC-correlations and comparison with the corresponding published data of structural related compounds [23,24]. Therefore, **2** was finally identified as 23-hydroxy-3α-[(*O*-*α*-L-^1^C_4_-rhamnopyranosyl-(1"→4')-*O*-*α*-L-^4^C_1_-xylo-pyranosyl)oxy]-olean-12-en-28-oic acid *O*-*α*-L-^1^C_4_-rhamnopyranosyl-(1'''''→4'''')-*O-β*-D-^4^C_1_-glucopyranosyl-(1''''→6''')-*O-β*-D-^4^C_1_-glucopyranosyl ester. 

**Table 1:** ^1^H- and ^13^C-NMR data of aglycones in **1** and **2** (500/125 MHz, pyridine-*d_5_*)**.**

No	1		2	
	δ _C_	δ _H_		δ _C_
**1**	38.9		39.0	
**2**	26.4		25.9	
**3**	85.1	3.95 *br s*	79.9	4.26 *br s*
**4**	39.1		43.2	
**5**	56.5		46.9	
**6**	18.8		17.2	
**7**	34.1		32.4	
**8**	40.1		39.6	
**9**	48.2		47.8	
**10**	37.4		36.5	
**11**	23.6		23.5	
**12**	123.3	5.70 *br s*	121.7	5.42 *br s*
**13**	144.4		143.5	
**14**	42.4		41.6	
**15**	27.6		27.9	
**16**	22.9		23.3	
**17**	47.3		47.4	
**18**	41.9	3.49 *dd* (13.5,3.0)	41.1	3.16 *dd* (13.5, 3.5)
**19**	46.5		46.9	
**20**	29.0		30.4	
**21**	34.1		33.7	
**22**	33.4		32.2	
**23**	28.5	0.91	63.6	4.11 *d* (10.5)
				3.75 *d* (10.5)
**24**	17.4	1.08 *s*	13.6	1.08 *s*
**25**	15.9	1.22 *s*	15.8	0.97 *s*
**26**	17.5	0.99 *s*	17.2	1.12 *s*
**27**	25.2	1.34 *s*	25.7	1.16 *s*
**28**	176.0		175.5	
**29**	31.0	0.89 *s*	32.7	0.85 *s*
**30**	24.0	0.91 *s*	23.3	0.87 *s*

δ in ppm and *J* values (Hz), were given in parentheses; All carbon and proton resonances were assigned on the basis of 2D (^1^H-^1^H COSY, HSQC and HMBC).

**Table 2:** ^1^H-, ^13^C-NMR and HMBC spectral data of sugar moieties in **1** and **2** (500/125 MHz, Pyridine-*d_5_*)**.**

No	1			2		
	δ _C_	δ _H_	HMBC cross peaks	δ _C_	δ _H_	HMBC cross peaks
**1'**	103.0	5.09 *br s*	C-3, 3'	104.1	5.12 *d* (7.5)	C-3, 3'
**2'**	73.0	4.94 ^a^	C-4'	73.8	4.71 *dd* (9.0, 7.5)	C-4'
**3'**	72.8	4.80 *dd* (9.0,3.0	C-1',5'	75.4	4.32 a	C-1',5'
**4'**	74.1	4.61 *t-like* (9.5)	C-2', 6'	78.4	4.63 m	C-2', 1''
**5' a**	70.6	4.58 *m*	C-3'	65.3	3.65 a	C-3'
**5' b**			C-3'		4.28 *br d* (10.5)	C-3'
**6'**	19.0	1.72 *d* (6.2)	C-4'	----	----	----
**1''**	95.9	6.50 *d* (8)	C-28, 3''	102.5	5.86 *br s*	C-4', 3''
**2''**	75.6	4.20 ^a^	C-4''	72.2	4.42 ^a^	C-4''
**3''**	78.8	4.47 *t-like* (10.5)	C-1'', 5''	72.2	4.67 ^a^	C-1'', 5''
**4''**	71.2	4.55 *t-like* (10.5)	C-2'', 6''	73.5	4.55 *t-like* (10.5)	C-2'', 6''
**5''**	78.5	4.40 *m*	C-3''	70.0	4.57 *m*	C-3''
**6'' a**	69.5	4.72 ^a^	C-4'', 1'''	18.2	1.66 *d* (6.5)	C-4''
**6'' b**		4.51 ^a^	C-4'', 1'''			
**1'''**	105.1	5.25 *d* (7.8)	C-6'', 3'''	95.5	6.27 *d* (8)	C-28, 3'''
**2'''**	74.3	4.35 ^a^	C-4'''	75.0	3.94 *dd* (8, 9.5)	C-4'''
**3'''**	76.8	4.44 ^a^	C-1''', 5'''	77.9	3.72 *t-like* (9.5)	C-1''', 5'''
**4'''**	79.0	4.64 *t-like* (10.5)	C-2''', 6'''	70.5	4.73 *t* (9)	C-2''', 6'''
**5'''**	77.4	4.39 *m*	C-3'''	77.7	4.01 *m*	C-3'''
**6''' a**	61.6	4.41 ^a^	C-4'''	69.4	4.95	C-4''', 1''''
**6''' b**		4.38 ^a^	C-4'''		4.69 ^a^	C-4''', 1''''
**1''''**				104.8	4.98 *d* (7.8)	C-6''', 3''''
**2''''**				74.4	4.08 ^a^	C-4''''
**3''''**				76.2	4.22 ^a^	C-1'''', C-5''''
**4''''**				79.7	4.59 *t-like* (9.5)	C-2'''', 6'''', 1'''''
**5''''**				76.8	4.17 *m*	C-3''''
**6'''' a**				60.8	4.15	C-4''''
**6'''' b**					4.35 ^a^	C-4''''
**1'''''**				101.3	6.29 *br s*	C-4'''', 3'''''
**2'''''**				72.0	4.67 ^a^	C-4'''''
**3'''''**				72.2	4.65 ^a^	C-1''''', 5'''''
**4'''''**				73.7	4.30 ^a^	C-2''''', 6'''''
**5'''''**				69.0	4.38 *m*	C-3'''''
**6'''''**				18.2	1.71 *d* (6.5)	C-4'''''

^a^ Unresolved proton resonances, δ in ppm and *J* values (Hz), were given in parentheses; All carbon and proton resonances were assigned on the basis of 2D (^1^H-^1^H COSY, HSQC and HMBC).

**Figure 3:**
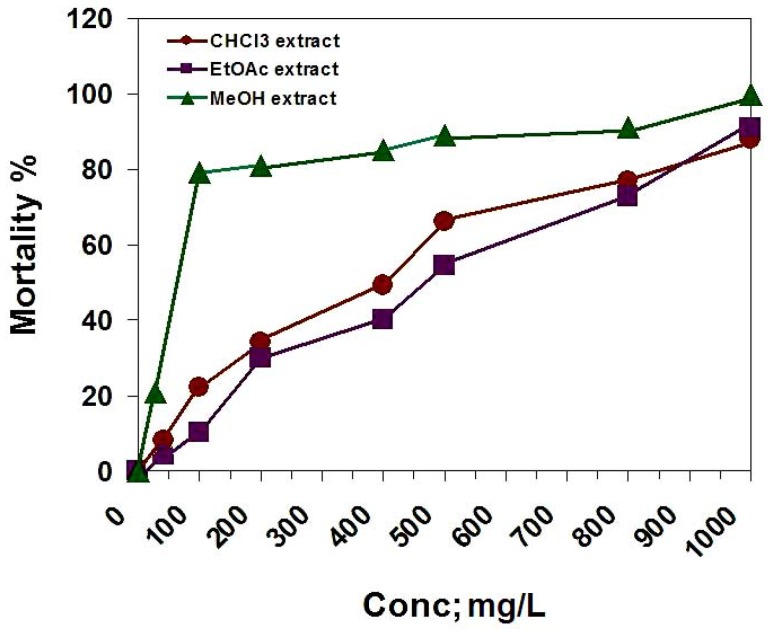
The cytotoxic activity of *D. repens* CHCl_3_, EtOAc and MeOH extracts against brine shrimp (*A. salina*).

In the brine shrimp lethality bioassay, the chloroform, ethyl acetate and methanol extracts were tested. The LC_50_ = 400, 470 and 64.3 mg/L, respectively (Figure 3). According to the standards of the National Cancer Institute (NCI), ED_50_ ≤ 20 μg/mL for impure compounds are considered to be active [25], so we assumed the median lethal concentration (LC_50_) as 200 ppm. According with this value, only the methanol extract is toxic on *A. salina*. 

**Figure 4:**
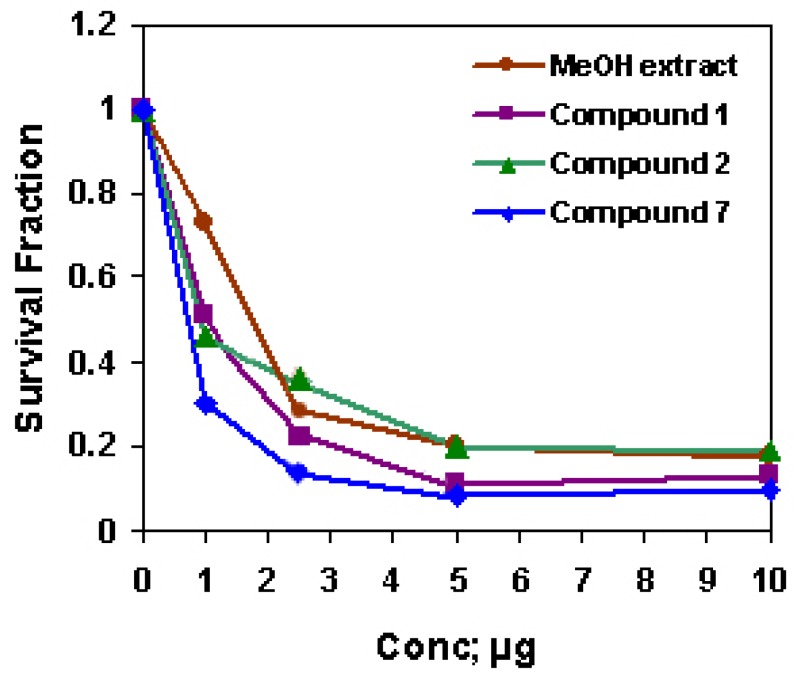
The cytotoxic activity of *D. repens* MeOH extract, **1**, **2** and **7** against HepG2 cell line.

The methanol extract and compounds **1**, **2** and **7** were cytotoxic for HepG2 cells and **7** was the most cytotoxic agent (IC_50_ = 1.68, 1.07, 0.94 and 0.60, respectively, Figure 4). Although different extracts of *D. repens have* been investigated as antioxidant and antiviral [9,10], to our knowledge, this work is the first trial to investigate the cytotoxicity of the methanol extract of *D. repens* leaves against HepG2 solid tumor cell lines. The strong anti-cancer activity of *D. repens* leaf methanol extract may be attributed to the corresponding activities of the extract constituents. There is a lack in the biological studies on the *Z*-form and on the mixed *E/Z*-form of acteoside, while the *E*-form was regularly investigated for different biological activities. *E*-acteoside is a phenylethanoid glycoside and is reported to be a strong antioxidant [26], protector from induced lipid peroxidation [27], and hepatotoxic [28], that was attributed to the large number of phenolic hydroxy groups. Recently, *E*-acteoside was found to have anti-inflammatory activity [29,30] and to possess anti-proliferative activity against B16F10: murine melanoma cells [7], A7r5: rat aortic smooth muscle cells [31], and HL-60: promyelocytic leukemia cells [32]. Some studies indicated that the antiproliferative activity of acteoside is associated with an induced apoptosis [32]. Many investigators attempted to structurally correlate the anti-cancer property of acteoside. Compared with their methanolysis products, it is suggested that the 3,4-dihydroxy-phenethyl alcohol group might be more responsible for their activities than the caffeoyl group [7,31] suggested that hydroxy groups of the aromatic rings appear to play a role in the anticancer effect of acteoside. The additional *Z*-caffeoyl group may enhance its cytotoxic properties to HepG2 cells. Since **7** is a mixture of two stereoisomers, its potential cytotoxic activity may be due to a separate role of the *Z*-form or to a synergistic effect between both of the geometrical isomers using the benefit of their variable steric configuration. Many bidesmosidic oleanane type triterpene saponins were reported to have a cytotoxic activity [33]. The tumor-specificity of the cytotoxic action seems to be influenced by the structure of the sugar portion of the saponins [34]. Although activities of **1** and **2** were moderate, it was reported that the aglycone with four sugar units exhibited greater available cytotoxicity than that, which possessed three sugars [35]. The results suggest that the presence of an additional sugar moiety of the oleanane- type saponins plays a role in mediating cytotoxicity.

## 3. Experimental

### 3.1. General

The NMR spectra for known compounds were recorded at 300 and 400 (^1^H) and 75, 100 MHz (^13^C) on a Varian Mercury 300 and Bruker APX-400 instrument, while new compounds were recorded at 500 (^1^H) and 125 MHz (^13^C) on a JEOL GX-500 NMR spectrometer and δ values are reported in ppm relative to TMS in the convenient solvent. IR spectra were measured on a Perkin Elmer FT-IR system spectrometer as KBr pellets. HRESI-MS analyses were run on a LTQ-FT-MS spectrometer (Thermo Electron, Germany). UV analyses of pure samples were recorded, separately, in MeOH solns. and different diagnostic UV shift reagents on a Shimadzu UV 240 spectrophotometer. Optical rotation values were measured on an ATAGO POLAX-D, No. 936216 (AEAGO Co., LTD., Japan) polarizer- with a 1 dm cell (ATAGO 901048). For column chromatography (CC), Sephadex LH-20 (Pharmacia, Uppsala, Sweden) and polyamide S (Fluka) were used. For paper chromatography Whatman No. 1 sheets (Whatman Ltd., England) were used, while silica gel G powder was used for saponin CC, and F_254_ for TLC (Merck, Germany).

### 3.2. Plant material

Leaves of *Duranta repens* were collected from plants growing in El-Orman Botanical Garden, Giza, Egypt in January 2005. The plant was authenticated by Dr. Wafaa M. Amer, Department of Botany, Faculty of Science, Cairo University, Giza, Egypt. Voucher specimens (Reg. No.: D–1) are kept in the herbarium, Medicinal Chemistry Department, Theodor Bilharz Research Institute, Giza, Egypt.

### 3.3. Cytotoxic assay

HepG2 cell line was obtained from National Cancer Institute, Kaser El Ainy St. Cairo, Egypt , eggs of *Artemia salina* (Artemia Inc., California) and saline artificial sea (Instant Oceanic, Marineland Labs, USA).

### 3.4. Extraction and isolation

The air dried powdered leaves of *D. repens* (900 g) were exhaustively extracted with 85 % MeOH (3 x 1.5 and 3 x 2.5 L, respectively), under reflux (70 °C). After evaporation of the solvent, the concentrated residue was defatted with light petroleum (60-80 ^o^C) to give the crude extract which was dissolved in water and the water-insoluble residue was removed by filtration. The water soluble portion was desalted by precipitation with excess MeOH to give a dry brownish residue (65 g) that was suspended in H_2_O and fractionated on a polyamide column (Ø 6.0 x 150 cm, 300 g). Elution was started with H_2_O followed by gradual increases of MeOH. Elution with H_2_O gave rise to two frs. I (2 L) and II (3 L). Elution with 20% MeOH afforded frs. III (3.5 L), IV (3 L) and V (3 L). Elution with 40-80% MeOH afforded frs. VI (2.5 L) and VII (3 L). Finally, elution with MeOH resulted in fr. VIII (3 L). On the basis of comp-TLC and PC with the use of UV light, 10% H_2_SO_4_ and Naturstoff spray reagents, the individual 92 fractions (250 ml each) were collected into eight collective fractions. Frs. I and II were found to be dark brown sugar material of no phenolic characters. A mixture of frs. III, IV and V, (20%) gave negative reaction with FeCl_3_ but pink color with sulphuric acid spray reagent on TLC when heated at 120 °C for three min. Collective fr. (III-V) was dried under vacuum (4.5 g) and subjected to separation over silica gel (Sigma, 28-200 mesh) column (Ø 3.0 x 50 cm, 100g) using a gradient of CHCl_3_-MeOH (9:1, 8:2, 7:3, 1:1, 3:7 and 0:1, each 75 ml) to give four main fractions (A-D) according to the differences in composition indicated by TLC analysis. Fr. A eluted with (CHCl_3_–MeOH 8:2, 2 g) was purified by silica gel column, eluted with CHCl_3_-EtOAc (8:2) to give pure **3** (75 mg). Fr. B was chromatographed on Sephadex and eluted with MeOH, whereby pure **4** (20 mg) was isolated. Crude **2** was crystallized from fr. C (CHCl_3_–MeOH, 1:1) and purified by repeated crystallization from MeOH to yield pure **2** (120 mg). Fr. D (CHCl_3_-MeOH, 3:7), was chromatographed on a silica gel column eluted with CHCl_3_-MeOH-H_2_O (3:7:0.1) to give pure **1** (110 mg). Fr. VI (40-60% MeOH, 950 mg) was subjected to repeated CC on cellulose and Sephadex with 20-60% aq. MeOH as an eluent, resulting in pure **5** (48 mg), **6** (52 mg) and **7** (100 mg). Fr. VII (60-80% MeOH, 750 mg) was fractionated on a Sephadex column using 30-70% aq. EtOH to give two main subfractions. Each subfraction was then separately chromatographed on Sephadex with MeOH for elution, whereby pure **8** (30 mg) and **9** (31 mg) were isolated. Fractionation of fr. VIII (2 g) on Sephadex with aq. EtOH 80% yielded two subfractions. The 1^st^ subfraction was fractionated on Sephadex using *n*-BuOH saturated with water for elution to give pure **10** (30 mg) and **11** (50 mg). Repeated CC of the 2^nd^ subfraction on Sephadex with EtOH for elution, afforded pure **12** (39 mg). All separation processes were followed up by Comp-TLC using solvent systems: MeOH-CHCl_3_ (11:1), MeOH-CHCl_3_ (3:7), S_3_: MeOH-EtOAc-CHCl_3_-H_2_O (35:32:28:2), S_4_: CHCl_3_-MeOH-H_2_O (65:35:3) and S_5_: *n*-BuOH- HOAc-H_2_O (4:1:1) or 2D-PC and Comp-PC using Whatman No. 1 paper with S_1_: *n*-BuOH-HOAc-H_2_O (4:1:5, top layer) and S_2_: 15% aq. HOAc as solvent systems. All aglycones and sugars obtained by acid and alkaline hydrolysis were identified by Comp-TLC and PC with authentic samples, using the previously described solvent systems, in addition to EtOAc-C_5_H_5_N-H_2_O (12:5:4), and specific spray reagents (e.g. vanillin HCl and aniline hydrogen phthalate).

### 3.5. Acid hydrolysis of ***1*** and ***2***

Ten mg of **1** and **2** were separately hydrolyzed with 2N HCl in EtOH under reflux at 90 °C for 3 h. The solvent was then evaporated until most of EtOH eliminated, the residue diluted with H_2_O (15 mL) and neutralized with NaHCO_3_, followed by extraction with CHCl_3_ (100 mL). The sapogenin was identified in CHCl_3_ layer by Comp-TLC (MeOH-CHCl_3_, 1.5: 9.5). The H_2_O-layer was then concentrated and subjected to Comp-PC (EtOAc-C_5_H_5_N-H_2_O, 12:5:4) against authentic sugar samples. 

### 3.6. Alkaline hydrolysis of ***1*** and ***2***

About 10 mg of **1** and **2** were separately refluxed with 1 M NaOH (10 mL) for 3 h. The hydrolysate mixture was neutralized and extracted with *n-*BuOH to give prosapogenin, which was subjected to acid hydrolysis. The sapogenin was extracted with CHCl_3_ and the H_2_O-layer was then concentrated and subjected to Comp-PC (EtOAc-C_5_H_5_N-H_2_O, 12:5:4) against authentic sugar samples. 

### 3.7. Durantanin IV *(**1**)*

Off-white amorphous powder; *R*_f_-value 0.53 (S_3_)**;** [α]**^25^_D_**: -2.9° (*c* 0.83, MeOH); IR (KBr), *ν*_max_ cm^-1^: 3,410 (s, OH), 2,800, 1,225 (S-O), 1,050; Negative HRESI-MS: *m/z* 1005.4732 [M-H]^-^ (calcd.: 1005.4728 for C_48_H_78_O_20_S, 601.4103 [M-H-deoxysulfodihexosyl]^-^, 455.3524 [M-H-deoxydeoxy-rhamnosyl]^-^ = [aglycone-H]^-^; ^1^H- and ^13^C-NMR spectral data reported in Table 1 and Table 2.

### 3.8. Durantanin V *(**2**)*

Off-white amorphous powder; *R*_f_-value 0.46 (S_5_)**;** [α]**^25^_D_**: -1.6° (*c* 0.2, MeOH); Negative HRESI-MS: *m/z* 1219.6117 [M-H]^-^ (calcd.: 1219.6131 for C_59_H_96_O_26_, 749.4180 [M-H-deoxyrhamnosyl-2hexosyl]^-^, 603.4282 [M-H-deoxyrhamnosyldihexosyl-deoxyrhamnosyl]^-^, 471.4425 [M-H-deoxyrhamnosyl-dihexosyldeoxyrhamnosylpentoside]^-^ = [aglycone-H]^-^; ^1^H- and ^13^C-NMR spectral data reported in Table 1 and Table 2. 

### 3.9. Brine shrimp lethality bioassay

Eggs of *Artemia salina* were allowed to hatch into their larvae [36]. The dried chloroform, ethyl acetate and methanol extracts of *D. repens* were separately dissolved in distilled water to give four assay concentrations (1000, 500, 100 and 10 mg mL^-1^). Solubility was aided by Tween 80 and each dose was examined in triplicate. Potassium dichromate was used as a reference drug and dissolved in seawater, to obtain concentrations of 1000, 100 and 10 µg mL^-1^. Assays were performed in test tubes with ten larvae each and the final volumes were adjusted to 5 mL sea salt soln. immediately after adding the shrimps. After 24 h, the number of surviving shrimps at each dose was recorded. The LC_50_ values were calculated by the use of the Instate computer program.

### 3.10. Measurement of potential cytotoxicity by SRB assay

Potential cytotoxicity of the methanol extract of *D. repens* leaves and the isolated compounds **1**, **2** and **7** were tested at the National Cancer Institute, Egypt using the method of [37]. Cells were plated in a 96-well plate (104 cells/well) for 24 h before treatment to allow the attachment of cells to the wall of the plate. Different concentrations of the fractions under investigation (0, 1, 2.5, 5 and 10 μg/mL) were added to the cell monolayer. Triplicate wells were prepared for each individual dose and they were incubated for 48 h at 37 °C in 5% CO_2_. After 48 h cells were fixed, washed and stained with sulforhodamine B stain. Excess stain was washed with acetic acid and attached stain was recovered with Tris-EDTA buffer and the color intensity was measured in an ELISA reader. The survival curve of the tumor cell line was plotted for each tested fraction.

## 4. Conclusions

Saponins isolated in this study were consistent with other reports that *Duranta repens* saponins were comprised of oleanane-type triterpenes [3,38,39]. The aglycone moiety in durantanin I-III is 2,16,23-tri hydroxyoleanolc acid (polygalactic acid). Thus, the finding of oleanane-type triterpene saponin may provide a significant chemotaxonomic proof for the *Duranta repens*. 

In the course of our studies, the brine shrimp lethality assay actually has proven to be a convenient system for monitoring biological activities of several *D. repens* extracts. Out of the several extracts screened for toxicity against the brine shrimp, the MeOH extract showed LC_50_ values less than 100 mg/L. These interesting results lend the authors for further supporting detailed phytochemical and biological studies.

## References

[1] Anis I., Anis E., Ahmed S., Mustafa G., Malik A., Amtul Z., Atta-ur-Rahman. (2001). Thrombin inhibitory constituents from *Duranta repens*. Helv. Chim. Acta.

[2] Ganapaty S., Jaya B.G., Naidu K.C. (1997). Phytochemical studies of roots of *Duranta repens*. Indian J. Nat. Prod..

[3] Hiradate S., Yada H., Ishii T., Nakajima N., Ohnishi-Kameyama M., Sugie H., Zungsontiporn S., Fujii Y. (1999). Three plant growth inhibiting saponins from *Duranta repens*. Phytochemistry.

[4] Bircher W.H. (1960). Gardens of Hesperides.

[5] Takeda Y., Morimoto Y., Matsumoto T., Ogimi C., Hirata E., Takushi A., Otsuka H. (1995). Iridiod glucosides from the leaves and stems of *Duranta erecta*. Phytochemistry.

[6] Rimpler H., Timm H. (1974). Iridiods and ecdysones from Verbenaceae. V. Iridiods from *Duranta repens* L. Z Naturfosch C.

[7] Nagao T., Abe F., Okabe H. (2001). Antiproliferative constituents in the plants 7. Leaves of *Clerodendron bungei* and leaves and bark of *C. trichotomum*. Biol. Pharm. Bull..

[8] Anis I., Ahmed S., Malik A., Yasin A., Choudary M.I. (2002). Enzyme inhibitory constituents from *Duranta repens*. Chem. Pharm. Bull..

[9] Shahat A.A., Nazif N.M., Abou Setta L.M., Ibrahim N., Vlitinck A.J. (2005). Phytochemical investigation and antioxidant activity of *Duranta repens*. Phytother. Res.

[10] Abou-Setta L.M., Nazif N.M., Shahat A.A. (2007). Phytochemical Investigation and Antiviral Activity of *Duranta repens*. J. .Appl. Sci. Res..

[11] Salama O.M., Amer M.M., Lahloub M.F., Spengel S. (1992). Repennoside, A new iridiod glucoside from *Duranta repens* Fruits, Mans. J. Pharm. Sci..

[12] Subramanian S.S., Nair A.G.R. (1972). Scutellarein and pectolinarigenin from the leaves of *Clerodenron phlomides* and *Duranta repens*. Phytochemistry.

[13] Nie R.L., Morita T., Kasai R., Zhou J., Wu C.Y., Tanaka O. (1984). Saponins from Chinese medicinal plants, (1). Isolation and structures of hemslosides. Planta Med..

[14] Imakura Y., Shigeru X., Kobayashi S., Mima A. (1985). Bitter phenylpropanoid glycosides from *Campsis chinensis*. Phytochemistry.

[15] Kobayashi H., Karasawa H., Miyase T., Fukushima S. (1985). Studies on the constituents of *Cistanchis Herba*. V. Isolation and structures of two new phenylpropanoid glycosides, cistanoside E and F. Chem. Pharm. Bull..

[16] Agrawal P.K., Agrawal P.K., Bansal M.C. (1989). Flavonoid Glycosides. Studies in Organic Chemistry 39,Carbon-13 NMR of Flavonoids.

[17] Sanchez-Contreras S., Diaz-Lanza A.M., Bartolome C., Bernabe M. (2000). Minor sulfated saikosaponins from the aerial parts of *Bupleurum rigidum* L. Phytochemistry.

[18] Mahato S.B., Kundu A.P. (1994). Review article number 98 ^13^C-NMR spectra of pentacyclic triterpenoids – a completion and some salient features. Phytochemistry.

[19] Kostova I., Dincheva D., Rentschb G.H., Dimitrovb V., Ivanovaa A. (2002). Two New Sulfated Furostanol Saponins from *Tribulus terrestris*. Z. Naturforsch..

[20] Kawai H., Kuroyanagi M., Umehara K., Ueno A., Satake M. (1988). Studies on the saponins of *Lonicera japonica* Thunb. Chem. Pharm. Bull..

[21] Schenkel E.P., Werner W., Schulte K.E. (1991). Die Saponine aus *Thinouia coriacea*. Planta Medica.

[22] Mshvildadze V., Elias R., Faure R., Rondeau D., Debrauwer L., Dekanosidze G., Kemertelidze E., Balansard G. (2004). Triterpenoid saponins from leaves of *Hedera pastuchowii*. Chem. Pharm. Bull..

[23] Mimaki Y., Kuroda M., Yokosuka A., Harada H., Fukushima M., Sashida Y. (2003). Triterpenes and triterpene saponins from the stems of *Akebia trifoliate*. Chem. Pharm. Bull..

[24] Shao C.J., Kasai R., Xu J.D., Tanaka O. (1989). Saponins from roots of *Kalopanax septemlobus* (Thunb.) Koidz., Ciqiu: Structures of Kalopanax-saponins C, D, E and F. Chem. Pharm. Bull..

[25] Cordell G.A., Kinghorn A.D., Pezzuto J.M., Colegate S.M., Molyneux R.D. (1993). Separation, structure elucidation and bioassay of cytotoxic natural products. Bioactive Natural Products: Detection, Isolation and Structural Identification.

[26] Ono M., Morinaga H., Masuoka C., Ikeda T., Okawa M., Kinjo J., Nohara T. (2005). New bisabolane-type sesquiterpenes from the aerial parts of *Lippia dulcis*. Chem. Pharm. Bull..

[27] Chiou W.F., Lin L.C., Chen C.F. (2004). Acteoside protects endothelial cells against free radical-induced oxidative stress. J. Pharm. Pharmacol..

[28] Lee K.J., Woo E.R., Choi C.Y., Shin D.W., Lee D.G., You H.J., Jeong H.G. (2004). Protective effect of acteoside on carbon tetrachloride-induced hepatotoxicity. Life Sci..

[29] Diaz A.M., Abad M.J., Frenandez L., Silvan A.M., De Santos J., Bermejo P. (2004). Phenylethanoid glycosides from *Scrophularia scorodonia*: in vitro anti-inflammatory activity. Life Sci..

[30] Lee J.Y., Woo E.R., Kang K.W. (2005). Inhibition of lipopolysaccharide-inducible nitric oxide synthase expression by acteoside through blocking of AP-1 activation. J Ethnopharmacol..

[31] He Z.D., Huang Y., Yao X., Lau C.W., Law W.I., Chen Z.Y. (2001). Purification of phenylethanoid glycosides from *Brandisia hancei* and the antiproliferative effects on rat aortic smooth muscle. Planta Med..

[32] Inoue M., Sakuma Z., Ogihara Y., Saracoglu I. (1998). Induction of apoptotic cell death in HL-60 cells by acteoside, a phenylpropanoid glycoside. Biol. Pharm. Bull..

[33] Lee K.T., Sohn I.C., Park H.J., Kim D.W., Jung G.O. (2000). Essential moiety for antimutagenic and cytotoxic activity of hederagenin monodesmosides and bisdesmosides isolated from the stem bark of *Kalopanax pictus*. Planta Med..

[34] Kuroda M., Mimaki Y., Hasegawa F., Yokosuka A., Sashida Y., Sakagami H. (2001). Steroidal glycosides from the bulbs of *Camassia leichtlinii* and their cytotoxic activities. Chem. Pharm. Bull..

[35] Huang H.C., Liaw C.C., Zhang L.J., Ho H.U., Kuo L.M.Y., Shen Y.C., Kuo Y.H. (2008). Triterpenoidal saponins from *Hydrocotyle sibthorpioides*. Phytochemistry.

[36] Fatope M.O., Ibrahim H., Takeda Y. (1993). Screening of Higher Plants Reputed as Pesticides Using the Brine Shrimp Lethality Assay. Int. J. Pharmacog..

[37] Skehan P., Storeng R., Scudiero D., Monks A., McMahon J., Vistica D., Warren J.T., Bokesch H., Kenney S., Boyd M.R. (1990). New colorimetric Cytotoxicity assay for anticancer drug screening. J. Nat. Cancer Inst..

[38] Kuo Y.H., Chen Z.S., Lin Y.L. (1996). Chemical components of the leaves of *Duranta repens* Linn. Chem. Pharm. Bull..

[39] Rao C.B., Rao T.N., Vikjayakumar E.K.S. (1978). Chemical examination of the fruits of *Duranta plumieri* Jacq. Indian J. Chem., B.

